# Improving medication adherence in chronic obstructive pulmonary disease: a systematic review

**DOI:** 10.1186/1465-9921-14-109

**Published:** 2013-10-20

**Authors:** Jamie Bryant, Vanessa M McDonald, Allison Boyes, Rob Sanson-Fisher, Christine Paul, Jessica Melville

**Affiliations:** 1 Priority Research Centre for Health Behaviour, University of Newcastle & Hunter Medical Research Institute, HMRI Building, University of Newcastle, Callaghan, NSW 2308, Australia; 2 Priority Research Centre for Asthma and Respiratory Disease, University of Newcastle & Hunter Medical Research Institute, HMRI Building, University of Newcastle, Callaghan, NSW 2308, Australia; 3 School of Nursing and Midwifery, University of Newcastle, Callaghan, NSW 2308, Australia

**Keywords:** Medication adherence, Medication compliance, Chronic obstructive pulmonary disease, Systematic review

## Abstract

Adherence to medication among individuals with chronic obstructive pulmonary disease (COPD) is suboptimal and has negative impacts on survival and health care costs. No systematic review has examined the effectiveness of interventions designed to improve medication adherence. Electronic databases Medline and Cochrane were searched using a combination of MeSH and keywords. Eligible studies were interventions with a primary or secondary aim to improve medication adherence among individuals with COPD published in English. Included studies were assessed for methodological quality using the Effective Practice and Organisation of Care (EPOC) criteria. Of the 1,186 papers identified, seven studies met inclusion criteria. Methodological quality of the studies was variable. Five studies identified effective interventions. Strategies included: brief counselling; monitoring and feedback about inhaler use through electronic medication delivery devices; and multi-component interventions consisting of self-management and care co-ordination delivered by pharmacists and primary care teams. Further research is needed to establish the most effective and cost effective interventions. Special attention should be given to increasing patient sample size and using a common measure of adherence to overcome methodological limitations. Interventions that involve caregivers and target the healthcare provider as well as the patient should be further explored.

## Introduction

### Global prevalence and burden of COPD

Chronic Obstructive Pulmonary Disease (COPD) is a progressive condition causing both pulmonary and systemic consequences [1,2]. The estimated global prevalence in adults aged 40 years and over is 9-10% [3]. Prevalence is projected to increase as a result of past high rates of tobacco use, and an ageing population [4,5]. In addition to significant healthcare costs, COPD causes a significant reduction in health-related quality of life [6,7].

### Optimal clinical management of COPD

Clinical practice guidelines recommend a multi-component approach for management of COPD [8]. This includes both oral and inhaled medications (e.g. bronchodilator therapy, corticosteroid therapy and combination therapy) and non-pharmacological interventions (e.g. pulmonary rehabilitation, lifestyle advice and self-management techniques) to slow disease progression, reduce exacerbations, and improve quality of life. Correspondingly, medication adherence is associated with reduced healthcare utilisation [9], significantly better survival [10,11], and overall better health outcomes [10,12,13]. However evidence suggests that adherence to medication is often sub-optimal.

### Medication adherence among patients with COPD

Non-adherence to medication in COPD is high, with adherence to inhaled and oral medications between 41.3% and 57% [14,15]. Contributing factors include medication type, prescribed dosing schedule, individual patient characteristics [15,16], and whether measurements to record adherence is direct (i.e. observations and blood serum measurements) or indirect (i.e. patient self-report, medication refill data and electronic records) [13]. Underuse of medications is most common [17], with up to 49.4% not taking nebulised treatments as prescribed [18-20]. Further, 31% employ ineffective inhaler dosing techniques [21] and more than 50% over-utilise medications during periods of respiratory distress [21].

### Factors predicting medication adherence

Two distinct patterns of behaviour are associated with medication non-adherence; intentional and unintentional [22-24]. *Intentional non-adherence* is the deliberate discontinuation or reduction in use of therapy during periods of symptom remission [25], often resulting from an erroneous understanding of the disease course and the goals of treatment [26]. *Unintentional non-adherence* occurs when patients do not adhere to treatment advice due to reasons out of their control [24], often relating to cognitive impairments, language barriers and physical disability. In the case of COPD, impaired vision or musculoskeletal problems affecting patient ability to use inhaled medications can be attributable [27]. The most commonly identified reason for unintentional non-adherence is complex medication regimes [28] and poly-pharmacy [29]. Multiple devices, poor awareness and understanding of the nature of COPD [30,31] confusion about prescribed medication regimes [25], and high rates of depression [14] have also been shown to negatively influence adherence.

### Strategies to improve medication adherence

Several reviews have examined the effectiveness of strategies including self-management, self-monitoring, and education in increasing medication adherence for a range of chronic conditions [32-35]. Few have found strategies which predictably produce large increases in adherence [35,36], however multi-dimensional interventions have been shown to produce the largest effects [37,38]. A recent review found more complex interventions that involved various combinations of information, reminders, self-monitoring, reinforcement, counselling, crisis intervention and supportive care to be the most effective, however also noted that many studies were not sufficiently powered to detect potentially clinically important effects [39]. A review by Rand and colleagues concluded very few published studies focus on adherence to treatment for COPD [40]. Given the high burden of COPD, strong evidence of poor adherence, and some differences in disease progression compared to other chronic conditions, there is a critical need to determine effective strategies to improve medication adherence.

This review aims to examine:

1. The methodological quality of publications examining the effectiveness of strategies to increase medication adherence for management of COPD;

2. The effectiveness of strategies to increase medication adherence.

## Methods

### Definitions

A number of terms have been used in the medical literature to describe health behaviour related to taking treatment. Adherence and compliance are often used interchangeably however these have different meanings which must be recognised. Medication *adherence* refers to the extent to which a patient’s behaviour in respect to their medication matches the recommendations of the prescriber [22,24,41]. The now largely obsolete term medication *compliance* refers to the extent to which a patient’s behaviour matches the prescriber’s advice [24]. Compliance is associated with negative connotations as it infers an authoritarian relationship where the prescribers’ role is to issue treatment and the patients’ role is to follow orders [22,24]. Therefore the term adherence has been used throughout this review.

### Literature search

The electronic databases Medline and the Cochrane Library were searched using the following MeSH terms: [Chronic Obstructive Pulmonary Disease OR Emphysema OR Pulmonary emphysema OR Chronic Bronchitis OR Obstructive Lung Diseases] AND [Medication adherence OR Patient Compliance; Compliance]; and the following keywords: [COPD] AND [patient adherence OR adherence]. Searches were not limited by publication year and restricted to English language human studies. The reference lists of all included intervention studies and identified reviews were also manually searched to identify any other relevant studies.

### Inclusion and exclusion criteria

Eligible studies were peer-reviewed intervention studies that examined medication adherence as a primary or secondary outcome measure in patients with a confirmed diagnosis of COPD. Studies that examined the timing, dosage, and frequency of medication were included. Papers related to adherence to oxygen therapy, exercise, pulmonary rehabilitation, inhaler device proficiency or self-management programs (without specific mention of medication adherence) were excluded. Letters, editorials, protocol papers, and case studies were also excluded. Papers examining medication adherence for other pulmonary diseases such as asthma were excluded, however studies which examined COPD together with other diseases were included where data for patients with COPD could be extracted separately to other pulmonary diseases. This criterion was applied given that COPD is a progressive disease, and that treatments for COPD differ markedly compared to other respiratory diseases [40].

### Data coding

Retrieved abstracts were initially assessed against the eligibility criteria by one author (JM) and rejected if the study did not meet inclusion criteria based on the title and abstract. The remaining studies were assessed against the inclusion criteria by two authors (JB and JM) and studies which met all criteria were retained. Of these, studies were assessed according to Effective Practice and Organisation of Care (EPOC) methodological criteria [42] and included if they fulfilled the design criteria. These included randomised controlled trials (RCT), controlled clinical trials (CCT), controlled before and after studies (CBA) and interrupted time series studies (ITS) [42]. Each study was assessed independently by two reviewers (JB and JM). Discrepancies were resolved through discussion between the reviewers. To assess intervention effectiveness, study data was extracted and included: aim of study; treatment type; study setting; sample characteristics (sample size, gender, age, diagnosis, smoking status); inclusion and exclusion criteria; intervention design; outcome measures; follow-up periods and study findings.

## Results

### Search results

**Figure 1:**
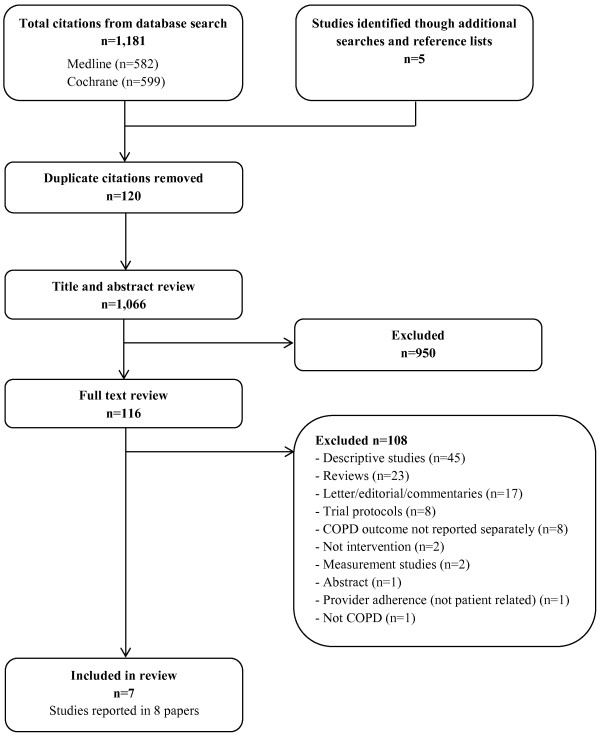
Flow chart of search strategy and study selection and assignment.

The initial search yielded 1,181 results with an additional five studies identified through manual searches of reference lists. After removing 120 duplicates, 1,066 unique papers were retained and assessed against eligibility criteria. Of these, seven separate studies reported in eight papers met criteria for inclusion in the review. A flow chart of the literature search and paper identification is provided in Figure 1.

### Study characteristics

The characteristics of the studies are reported in Additional file 1: Table S1. Three studies were conducted in the United States [43-46] with the remainder in Norway [47], Spain [48], Jordan [49] and the United Kingdom [50]. Adherence to various medications were examined including (i) Theophylline [43]; (ii) Prednisolone and β_2_ agonists [47]; (iii) Short and long-acting β_2_ agonists, anticholinergic, methylxanthines, inhaled and oral corticosteroids [48]; (iv) Short and long-acting β_2_ agonists, long acting anti-cholinergic inhaled steroids, oral steroids, antibiotics [49]; (v) Short and long-acting β_2_ agonist, long acting anticholinergic, inhaled steroids, oral steroids [50]; and (vi) Ipratropium bromide [44,45]. Medication adherence was the primary outcome in five studies (reported in six publications) [43-47,50], and a secondary outcome in two [48,49]. Outcomes were measured directly (blood serum ratios [43], observation of inhaler technique [48]) and indirectly (prescription refills [43], Medication Adherence Scale [48], Inhaler Adherence Scale [48], Moriksy Scale [46,49,50], inhaler device data [44,45], patient self-report [44], pharmacy data [47], canister weighing [44] and tablet counts [46]). Five studies assessed adherence using two or more measures [43-48]. Brief counselling [43], monitoring and feedback about medication use [44,45], and multi-component interventions were examined [44-50]; delivered by pharmacists [43,46,49,50], general practitioners [47], and a health educator [44,45]. Timing of outcome measures was relatively homogenous, with the majority involving a 6 and/or 12 month follow-up.

### Methodological quality

The methodological quality of included studies is reported in Additional file 2: Table S2. Low risk of bias was rated across all nine criteria in one study [48], and across seven and eight criteria in two studies [47,49]. The remaining studies had differing methodological limitations. There were four RCTs [46,48-50]. The remainder were CCTs as the method used for randomisation was inadequately detailed [43-45,47], such as concealment of allocation to groups [43-45,47], and limited clarity in whether baseline outcome measures were similar prior to the intervention [43-46]. Two studies were judged to have a high risk of bias as baseline demographic characteristics between groups were not reported [43-45]. One study was judged to have a high risk of bias for selective outcome reporting, as only a statement that there was no difference in medication adherence for patients with COPD was contained within the publication [46] and specific data and measures of statistical significance were not reported.

### Interventions that improved medication adherence

Additional file 1: Table S1 describes the characteristics of the included studies. Interventions were behavioural interventions including *brief counselling and monitoring and feedback*, and *multi-component interventions* combining *behavioural and educational approaches*. Five studies (reported in six publications) found positive results [43-45,48-50].

#### ***Brief counselling***

De Tullio and colleagues [43] examined the effectiveness of brief counselling compared to usual care on adherence to theophylline in a veteran population. Intervention participants received 3–5 minutes of counselling on theophylline and the importance of taking medication as recommended, delivered by a pharmacist following a routine physician visit. Counselling significantly improved adherence measured by drug serum levels at 4.5 months post-intervention, and prescription refills at 6 months post-intervention (*p* = .4).

#### ***Monitoring and feedback***

Nides and colleagues [44] examined the effectiveness of a Nebuliser Chronolog feedback system, as part of a larger 12 week smoking cessation Lung Study [51]. The Nebuliser Chronolog is a microprocessor device with the ability to record the date and time of each inhaler actuation. The intervention group received the Nebuliser Chronolog, were informed of the device capabilities and provided with printed feedback of their recordings after weeks one and seven. Participants also had brief sessions with a health educator who reviewed inhaler usage and provided individual feedback at each four month follow-up. The control group were given the Nebuliser Chronolog and were only informed the device recorded the amount of inhaled drug use. At the four month follow-up the intervention group showed significantly better adherence to the prescribed three sets of medication each day (*p* = .003), had greater proportion of adherent days (*p* < .0001) and had greater proportion of inhalations taken as prescribed (*p* < .0001) than the control group. Simmons and colleagues [45] reported the results from this study for the four month follow-up through to the 24 month follow-up. A significantly higher level of adherence was shown for the intervention group at each individual follow-up period (*p* < .05). Both groups showed a statistically significant difference between these intervals for every follow-up period.

#### ***Multi-component interventions***

Three studies examining multi-component interventions were effective. One of the most complex interventions examined an integrated self-management and co-ordination of care intervention in patients discharged from hospital after exacerbation of COPD [48]. Patients received (i) an individually tailored care plan at discharge, shared with their primary care team; (ii) a two-hour individual education session about self-management with written educational information; (iii) a visit by a nurse and primary care team within 72 hours of discharge; (iv) weekly phone calls from a specialised case manager nurse in the first month post-discharge, then phone calls at 3 and 9 months, as well as the option to phone the nurse if symptoms of COPD worsened. Adherence to inhaled treatments significantly increased at 12 months (intervention, 71%; control, 37%; *p* = .009), as well as correct inhaler use (*p* < .001). There were no differences between groups in adherence to oral treatments, but rates were already high with 85% adherence in the control group and 90% adherence in the intervention group at 12 months. Improvements in disease knowledge and nutritional status, and reductions in hospital admissions were also found.

Structured face-to-face motivational interviewing from a pharmacist at an outpatient clinic, the provision of a medication table, and education about symptom control, technique for expectoration and the importance of simple exercises had a positive impact on adherence to short and long acting β agonists, inhaled and oral steroids and antibiotics at 6 months follow-up (*p* < .05) [49]. Positive effects were also found for a more intensive individually tailored pharmacist-delivered intervention [50] involving a one-hour face-to-face education session on COPD, medications and breathing techniques; educational booklets on techniques for improving health status; a customised action plan; and telephone follow-up. Participants who smoked received motivational interviewing and were provided with a referral to hospital smoking cessation programs. There were significant improvements in medication adherence at 6 months (intervention, 81%; control, 63%; *p* = .019) and 12 months follow-up (intervention, 78%; control, 60%; *p* = .019).

### Interventions that did not improve medication adherence

Two studies found no significant effect on adherence [46,47]. Both used multi-component approaches.

Gallefoss and colleagues [47] examined the effectiveness of face-to-face education program and self-management plan compared to usual general practitioner care on adherence to Prednisolone and β_2_ agonists. Participants received an educational patient brochure, two 2-hour group sessions focusing on pathophysiology of disease, medications, symptom awareness and treatment plans, and either one or two 40-minute individual sessions delivered by a trained nurse and physiotherapist. An individual treatment self-management plan was provided at the final face-to-face session, and the patients’ personal understanding of the plan was discussed and tested. No significant differences in steroid inhaler adherence or use of oral steroids were found at 12 months follow-up. Overall adherence at 12 months was 50% in the intervention group, and 58% in the control group.

Solomon and colleagues [46] examined the effectiveness of a pharmaceutical care intervention in an ambulatory setting. The intervention group received face-to-face and telephone pharmaceutical care from a clinical pharmacist and pharmacy residents which included: (i) management of drug therapy; (ii) pharmacist and physician collaboration to implement patient-specific stepped care; (iii) education about COPD; (iv) counselling to address patient concerns; (v) patient assessment and care through clinic visits and telephone follow-up. The authors state that there were no significant differences in medication adherence between intervention and control groups at 6 months follow-up, however specific data were not reported.

## Discussion

Currently available pharmacotherapy for COPD can significantly reduce the frequency and length of exacerbations, reduce hospital admissions and therefore the cost of care, and slow disease progression [9,10]. However, medications are optimally effective when used as prescribed. This systematic review examined the effectiveness of interventions designed to improve medication adherence for individuals with COPD. Of the seven intervention studies identified, five found improvements in adherence at either short or long term follow-up periods [43,44,48-50].

### Methodological quality

Overall, methodological quality of studies included in the review was variable. Recurring limitations included inadequacies in describing randomisation methods, assessing similarities of baseline characteristics between groups, unblended assessment of primary outcome measures and failure to report study power. This compromises the strength of evidence.

Various measures of medication adherence were used which made it difficult to compare rates and effect of interventions across studies, precluding the aggregation of findings in meta-analysis. The lack of a gold-standard measure of medication adherence and the need for consensus about a uniform measure has been well documented [52,53], but is critical to resolve to move the evidence base in this area forward.

### Effectiveness of strategies to improve medication adherence

The majority of studies included in this review utilised multi-component strategies, however specific intervention components of success could not be determined. In addition, it would also be useful for future studies to explore issues of the relative costs and benefits of more intensive versus less intensive approaches given the increasing pressure on limited health care resources.

Studies that showed statistically significant intervention effects did not necessarily result in improved adherence. In one study for example, only 71% of the intervention group were adherent at 12 months follow-up and only 86% correctly used inhaler therapies [48]. Similarly in another study, despite structured face-to-face motivational interviewing increasing adherence compared to a control group, nearly one third of participants were still non-adherent at 6 months follow-up [49]. Achieving optimal adherence and determining which interventions achieve highest level of adherence is an important and ongoing issue. Our findings suggest further research is needed to identify effective strategies.

### Implications and directions for future research

The reasons for non-adherence among patients with COPD are multi-factorial and complex. Future research should further explore the multi-component interventions found effective in the two most rigorous studies included in the review [48,49]. Also, strategies demonstrating benefits for other chronic diseases, and directly address key problems that affect adherence for COPD, such as complex medication regimes, should also be further explored.

#### ***Shared decision making***

None of the studies described the use of shared decision making in their interventions. A person-centred approach to care where the agendas’ of the patient and health care provider are addressed and agreed upon may effectively improve adherence in COPD [54]. A recent study examined the effect of shared decision making compared to clinician decision making among patients with asthma, using adherence to controller therapy as the outcome [55]. In the shared decision making group, participants negotiated their treatment, had the opportunity to summarise treatment goals, and were provided with information on treatment and treatment options. Statistically significant improvements in adherence and clinical outcomes were demonstrated [55].

#### ***Interventions that target the physician***

Optimal medication use requires collaborative interventions that address patient barriers but also provider prescribing behaviour. All studies identified focused on patient education and counselling rather than system or provider-level interventions. Enhancing provider skills in patient education, communication, and adherence counselling has been explored in diseases such as asthma and resulted in improved satisfaction and health outcomes for patients [56]. Simplifying medication regime, wherever possible, should also be explored. Changes in dosing regimen to limit the number of doses required per day improved adherence for hypertension and cholesterol treatment [35]. No studies specified that they attempted to simplify the medication regime.

#### ***Involving caregivers***

A caregiver is generally defined as an unpaid individual that provides physical or emotional support [57]. A recent study demonstrated positive associations between having a caregiver (spousal or non-spousal) and adherence in COPD patients, with better adherence to antihypertensive and long acting β agonist medications, and less likely to smoke than those without [58]. No studies included in the review examined the influence of caregivers. Future intervention studies could explore this, including active encouragement to be part of the patients care during clinical appointments, and targeted education about the importance of medication adherence.

### Limitations of the review

Our findings should be considered in light of several limitations. Firstly, the review only identified publications in peer-reviewed journals. However, it is unlikely that rigorous studies finding a positive intervention effect exist in the unpublished literature. Secondly, meta-analysis was not possible given the wide variety of measures used to assess medication adherence. Finally, we included studies that evaluated interventions to improve medication adherence. We acknowledge the importance of non-pharmacological interventions in COPD such as physical activity, nutrition and other lifestyle recommendations, and that interventions that improve adherence to these treatment recommendations also require evaluation.

## Conclusions

Improving medication adherence among individuals with COPD is critical to optimising patient outcomes. There is a clear need for rigorous research to determine effective interventions for improving medication adherence for individuals with COPD. Future research should consider interventions that have been successful in other chronic diseases such those involving partnerships between patients and clinicians.

## Competing interests

Dr Vanessa McDonald has participated in educational symposia funded by AstraZeneca, GlaxoSmithKline and Novartis and has participated in studies funded by GlaxoSmithKline. The remaining authors have no competing interests to declare.

## Authors’ contributions

JB and RSF conceived the review concept. JB and JM undertook data extraction. All authors contributed to the drafting of the manuscript. All authors read and approved the final manuscript.

## Supplementary Material

Additional file 1 Characteristics of included studies (n = 8). Click here for file

Additional file 2 EPOC methodological quality ratings of included studies. Click here for file
