# Supplementary material for: Improving medication adherence in chronic obstructive pulmonary disease: a systematic review
Source: Respir Res. 2013 Oct 20;14(1):109. doi: 10.1186/1465-9921-14-109 (PMC4015036; doi:10.1186/1465-9921-14-109)
Supplement: Additional file 1 — Characteristics of included studies (n = 8). [file 1465-9921-14-109-S1.docx]

**Table One. Characteristics of included studies (n=8)**

| **Reference**  Country  Design | **Sample**  N; Age; Gender; Diagnosis;  Current smokers; Setting;  Medication types | **Eligibility**  Inclusion criteria; Exclusion criteria | **Intervention** | **Outcome measures**  **Follow-up time points** | **Findings** |
| --- | --- | --- | --- | --- | --- |
| **De Tullio et al., 1987[**[**43**](#_ENREF_43)**]**  USA  CCT | **N:** 60  **Age:** I: M=62.1 (SD=9.3); C: M=63.2 (SD=6.7).  **% Male:** I: 100; C: 100.  **Diagnosis:** COPD.  **% Current smokers:** I: 23; C:40.  **Setting:** Outpatient pulmonary clinic at a veteran’s medical centre.  **Medication types:** Theophylline. | **Inclusion criteria:** Theophylline therapy established for at least one month; dosing stabilised for at least one week; capable of taking own medication unassisted.  **Exclusion criteria:** None reported. | **Intervention:** N=30. Face-to-face 3-5 minute counselling intervention delivered by clinical pharmacist following routine visit with physician. Counselling included information about drug and the importance of taking medication as prescribed.  **Control:** N=30. No verbal instruction. Allowed to ask the clinical pharmacist questions regarding medication. | **Measures:** i) Ratio of actual serum theophylline level to that predicted by pharmacokinetic estimation (*a/p* ratio); ii) Number of prescription refills in 155 days.  **Follow up:** Serum levels assessed average of 4.5 months; 6 month prescription refill. | - I participants had significantly higher actual serum theophylline levels compared to C (*p*=.0001). - I participants had higher actual serum theophylline levels within ±10% of predicted levels (*p*=.0006) compared to C. - I participants had higher actual serum theophylline levels within ±20% of predicted levels (*p*=.04) compared to C. - I participants had significantly higher adherence as assessed by refill records M=4.8; SD=.94µg/ml compared to C: M=3.8; SD=1.24µg/ml; p=.05). |
| **Gallefoss and Bakke, 1999*[**[**47**](#_ENREF_47)**]**  Norway  CCT | **N:** 62  **Age:** I: M=57 (SD=9); C: M=58 (SD=10).  **% Male:** I: 52; C: 48.  **Diagnosis:** Bronchial asthma or COPD (mild–moderate).  **% Current smokers:** I: 38.7; C: 38.7.    **Setting:** Outpatient chest clinic, Norway.  **Medication types:** Prednisolone & β_2_ agonists. | **Inclusion criteria:** Bronchial asthma or COPD; aged 18-70 years; pre-bronchodilator FEV_1_ ≥40% and <80% of predicted.  **Exclusion criteria:** Suffering from serious disease (unstable coronary heart disease, heart failure, serious hypertension, diabetes mellitus, kidney or liver failure). | **Intervention:** N=31. Face-to-face education program and self-management plan. Program included: 19-page patient brochure with essential information on COPD, medication, self-assessment and self-management, two 2-hour group sessions on two separate days, 1 week apart; 1 or 2 40-min individual sessions delivered by trained nurse and physiotherapist. Individual treatment plan provided at final teaching. Personal understanding of plan discussed and tested.  **Control:** N=31. Usual care delivered by GP. | **Measures:** i) Defined daily dosages using Anatomical Therapeutic Chemical classification index; ii) Dispensed dosages reported monthly from pharmacy data registers; iii) Steroid inhaler compliance (SIC) (defined as SIC > 75% - dispensed /prescribed x 100).  **Follow up:** 1 year follow-up with GP. | - No significant difference in compliance (I: 50%; C: 58%; *p*=.56). - I participants were dispensed half the amount of short-acting inhaled β_2_-agonists (*p*=.03). - No difference in use of oral steroids (I: 69% vs. C: 44%; *p*=.07). |
| **Garcia-Aymerich et al., 2007[**[**48**](#_ENREF_48)**]**  Spain  RCT | **N:** 113  **Age:** I: M=72 (SD=10). C: M=73 (SD=9) (followed-up participants only).  **% Male:** I: 75; C: 93.  **Diagnosis:** COPD (FEV_1:_ M=1.2 (SD=.5).  **% Current smokers:** I: 20; C: 13.  **Setting:** Tertiary hospital clinic.  **Medication types:** Short-acting β_2_-agonists; long-acting β_2_-agonists; anticholinergic; methylxanthines; inhaled corticosteroids; oral corticosteroids. | **Inclusion criteria:** Admitted because of exacerbation requiring hospitalisation for >48 hours.  **Exclusion criteria:** Not living in healthcare area or living in a nursing home; lung cancer or other advanced malignancies; logistic limitations including extremely poor social conditions, illiteracy, no phone access at home; extremely severe  neurological or cardiovascular comorbidities. | **Intervention:** N=44. Assessment of the patient at discharge; 2 hour educational session on self-management including written information; possibility to phone nurse if symptoms worsened; joint visit by nurse and primary care team within 72 hours post-discharge; weekly phone call first month post-discharge and one phone call at 3 and 9 months.  **Control:** N=69. Usual care. | **Measures:** i) Medication Adherence Scale (MAS); ii) Inhaler Adherence Scale (IAS); iii) Observed skills for administration of inhaled drugs.  **Follow up:** 6 and 12 months. | - Significant difference in inhaled treatment adherence at 12 months (I: 71%; C: 37%; *p*=.009). - Significant difference in correct inhaler use (I: 86%; C: 24%; *p*≤.001). - No significant difference in adherence to oral treatment at 12 months (I: 90%; C: 85%; *p*=.57). |
| **Jarab et al., 2012[**[**49**](#_ENREF_49)**]**  Jordan  RCT | **N:** 133  **Age:** I: Median=61 (IQR=14); C: Median=64 (IQR=15).  **% Male:** I: 39.4; C: 41.8.  **Diagnosis:** COPD.  **% Current smokers:** I: 54.5; C: 56.7.  **Setting:** Outpatient hospital COPD clinic.  **Medication types:** Short-acting β-agonists; long-acting β-agonists;  long-acting anti-cholinergic; inhaled steroids; oral steroids; antibiotics. | **Inclusion criteria:** Attend outpatient COPD clinic; confirmed diagnosis by hospital consultant for >1 year; >35 years old; FEV_1_ of 30–80% of predicted; consultant agreement that patient suitable for trial.  **Exclusion criteria:** Moderate to severe learning difficulties; mobility problems; confusion; disorientation or terminal illness; congestive heart failure; attended pulmonary rehabilitation program or consulted pulmonary nurse or clinical pharmacist in last 6 months. | **Intervention:** N=66. Structured face-to-face motivational interviewing provided by clinical pharmacist at an outpatient clinic. Education included symptom control, technique for sputum expectoration and importance of simple exercises for physical activity. Clinical pharmacist completed medication table and provided take-home booklet. Referral to smoking cessation program.  **Control:** N=67. Usual care. | **Measures:** Morisky scale.  **Follow up:** 6 months. | - Significant difference in proportion of non-adherent patients in I (28.6%) compared to C (48.4%) at 6 months (*p*<.05). |
| **Khdour et al., 2009[**[**50**](#_ENREF_50)**]**  UK  RCT | **N:** 173  **Age:** I: M=65.63 (SD=10.1); C: M=67.3 (SD=9.2).  **% Male:** I: 44.2; C: 43.7.  **Diagnosis:** COPD.  **% Current smokers:** I: 20.9; C: 21.8.  **Setting:** Hospital based outpatient clinic.  **Medication type:** Short-acting β_2_-agonist; long-acting β_2_-agonist; long acting anticholinergic; inhaled steroids; oral steroids. | **Inclusion criteria:** Confirmed diagnosis of COPD by the hospital consultant for > 1 year; FEV_1_ of 30–80% of predicted normal value; >45 years old.  **Exclusion criteria:** Congestive heart failure; moderate to severe learning difficulties; attended a pulmonary rehabilitation program in the last 6 months; severe mobility problems; terminal illness. | **Intervention:** N=86. i) Assessment of disease knowledge; smoking status; medication adherence; self-efficacy in managing breathing difficulty; exercise and diet habits conducted by researchers and results forwarded to clinical pharmacist to allow tailoring of intervention; ii) One hour face-to-face education delivered by clinical pharmacist on disease state, medications and breathing techniques. Patients given booklets and a customised action plan. Motivational interviewing provided to participants who smoked, and referral to hospital smoking cessation program made. At outpatient clinic visits (every 6 months) participants received reinforcement of education by clinical pharmacist, as well as telephone calls at 3 and 9 months.  **Control:** N=87. Usual care. | **Measures**: Morisky Scale.  **Follow up:** 6 and 12 months. | - At 6 months follow-up, significantly higher adherence to medication in I (81%) compared to C (63%); *p*=.019. - At 12 months follow-up, significantly higher adherence to medication in I (77.8%) compared to C (60%); *p*=.019. |
| **Nides et al., 1993[**[**44**](#_ENREF_44)**]**  USA  CCT | **N:** 251  **Age:** I: M=49 (SD=6.4); C: M=50.3 (SD=6.3).  **% Male:** I: 63; C: 56.  **% Current smokers:** I: 100; C: 100.  **Diagnosis**: COPD.  **Setting:** University of California and Johns Hopkins University centres.  **Medication type:** Ipratropium bromide or placebo. | **Inclusion criteria:** Aged 35-60 years; active cigarette smokers; spirometric evidence of mild to moderate airflow obstruction as indicated by FEV_1_/FVC of ≤70% and FEV_1_ of 55-90% of predicted.  **Exclusion criteria:** Serious health problems which might limit study participation or life expectancy; regular use of a bronchodilator or β-adrenergic blocking agent. | **Intervention:** N=140. Nebuliser chronolog (NC) provided to patients. Patients instructed about ability of the NC to record the time and date of each actuation. Provided with printed copies of own NC record at end of weeks 1 and 7 of the 12-week smoking cessation program. Health educator and participant jointly reviewed feedback about adherence (5 min sessions). Praise given for satisfactory use. Behavioural strategies such as anchoring inhaler use to daily routines were collaboratively developed to address problem areas. Brief feedback sessions continued at each 4-month follow-up visit.  **Control:** N=111. Patients provided with NC monitor and told monitor would record the amount of inhaled drug used. No feedback provided. | **Measures:** i) NC device data on number and intervals of actuations; ii) Self-reported adherence “how frequently on average are you using your inhaler at present” with seven response options ranging from “not at all” to “4 or more times per day”; iii) Inhaler canister weighing before being dispensed and at follow-up.  **Follow up:** 4 months. | - I participants adhered more closely to the prescribed three sets per day (M=1.95; SD=0.68) compared to C (M=1.63; SD=0.82); *p*=.003. - I participants had greater proportion of adherent days (M=60.2; SD=25.9) compared to C (M=40.4; SD=28.2); *p*<.0001. - I participants had greater proportion of actuations taken as prescribed (M=88.8; SD=9.6) compared to C (M=68.8; SD=25.7); *p*<.0001. - 28% of I participants had >80% adherent days compared to only 7.9% of C participants; *p*<.002. |
| **Simmons et al., 1996[**[**45**](#_ENREF_45)**]**  USA  CCT | **N:** 231  **Age:** I: M=50.3; C: M=48.4  **% Male:** Not reported.  **Diagnosis:** COPD.  **% Current smokers:** I: 100; C: 100.  **Setting:** University of California, Los Angeles and Johns Hopkins University.  **Medication type:** Ipratropium bromide. | **Inclusion criteria:** Aged 35-60 years; active cigarette smokers; spirometric evidence of mild to moderate airflow obstruction as indicated by FEV_1_/FVC of ≤70% and FEV_1_ of 55-90% of predicted.  **Exclusion criteria:** Serious health problems which might limit study participation or life expectancy; regular use of a bronchodilator or β-adrenergic blocking agent. | **Intervention:** N=129. Aware that inhaler had a nebuliser chronolog (NC) to record date and time of each use. Readings of actuation dates and times used to provide feedback at weeks 1 and 10 following their groups quit date and each 4 month follow-up.  **Control:** N=102. Not aware of ability of NC to record date and time, however aware that the NC would monitor total medication used. | **Measures:** NC device data examining: i) Mean number of daily sets of use (mean number of times inhaler is used each day) in two week interval following issue of NC and each subsequent follow-up visit; ii) Changes in mean number of sets per day (comparison of last 2 week period before the follow-up visit and first 2 week period after the follow-up visit).  **Follow up:** 4, 8, 12, 16, 20 and 24 months. | - I group had significantly greater mean number of daily sets of use at each follow up compared to C: 4 months- I: M=1.93 (SD=.69); C: M=1.6 (SD=0.83); *p*<.0035.  8 months- I: M=1.76 (SD=.83); C: M=1.31 (SD=.89); *p*=.0003.  12 months- I: M=1.74 (SD=.89); C: M=1.29 (SD=.91); *p*=.0007.  16 months- I: M=1.7 (SD=.89); C: M=1.27 (SD=.92); *p*=.0018.  20 months- I: M=1.56 (SD=.87); C: M=1.22 (SD=.97); *p*=.019. 24 months- I: M=1.65 (SD=.89); C: M=1.16 (SD=.95); *p*=.0006. - No significant differences between groups in mean number of sets per day from last 2 week period before follow-up and first 2 week period after follow-up. |
| **Solomon et al., 1998*[**[**46**](#_ENREF_46)**]**  USA  RCT | **N:** 98  **Age:** I: M=69.3 (SD=5.9); C: M=69.3 (SD=9.2).  **% Male** = I: 100; C: 100.  **Diagnosis:** COPD.  **% Current smokers:** Not reported.  **Setting:** 10 Department of Veterans Affairs medical centres and 1 academic medical centre.  **Medication type:** Not reported. | **Inclusion criteria:** ≥40 years; ambulatory patient; pulmonary function tests to diagnose COPD; currently receiving treatment that included ≥1 metered dose inhaler (MDI); mentally and physically able to use MDI/spacer inhaler; read and write English; understand study protocols; telephone access.  **Exclusion criteria:** History of mechanical ventilation in past year; life expectancy <6 months; hospitalised, visited ED, or lung infection in past 2 weeks; decompensated congestive heart failure (class 3 and 4); use of alcohol or drugs which would interfere with study; involvement in other drug trials in past 30 days or scheduled during study. | **Intervention:** N=43. Patient-centred pharmaceutical care provided face-to-face and via telephone by clinical pharmacist and pharmacy residents. Included: management of drug therapy; collaboration with physician to implement patient-specific stepped care; education about COPD; counselling to address patient concerns; patient assessment and care through clinic visits and telephone follow-up.  **Control:** N=55. Usual pharmacy care. | **Measures**: i) Morisky scale; ii) Tablet counts.  **Follow up:** 6 months. | - Authors state no significant difference in medication compliance. Data not reported. |
