# Supplementary material for: Improving medication adherence in chronic obstructive pulmonary disease: a systematic review
Source: Respir Res. 2013 Oct 20;14(1):109. doi: 10.1186/1465-9921-14-109 (PMC4015036; doi:10.1186/1465-9921-14-109)
Supplement: Additional file 2 — EPOC methodological quality ratings of included studies. [file 1465-9921-14-109-S2.docx]

**Table Two. EPOC methodological quality ratings of included studies.**

|  | **Design** | **Allocation sequence adequately generated?** | **Concealment of allocation?** | **Baseline outcome measurement similar?** | **Baseline characteristics similar?** | **Incomplete outcome data adequately addressed?** | **Knowledge of allocated interventions prevented?** | **Protection against contamination?** | **Selective outcome reporting?** | **Free from other risk of bias?** |
| --- | --- | --- | --- | --- | --- | --- | --- | --- | --- | --- |
| **De Tullio et al., 1987[43]** | CCT | **●** | **‡** | **‡** | ● | ■ | ■ | ● | ■ | ■ |
| **Gallefoss and Bakke, 1999[47]** | CCT | **‡** | **‡** | ■ | ■ | ■ | ■ | ■ | ■ | ■ |
| **Garcia-Aymerich et al., 2007[48]** | RCT | ■ | ■ | ■ | ■ | ■ | ■ | ■ | ■ | ■ |
| **Jarab et al., 2012[49]** | RCT | ■ | ■ | ■ | ■ | ■ | **‡** | ■ | ■ | ■ |
| **Khdour et al., 2009[50]** | RCT | ■ | ■ | ■ | **‡** | ■ | ● | ■ | ■ | ■ |
| **Nides et al., 1993[44]** | CCT | ● | **‡** | **‡** | ● | ■ | ■ | ● | ■ | ■ |
| **Simmons et al., 1996[45]** | CCT | ● | **‡** | **‡** | ● | ■ | ■ | ● | ■ | ■ |
| **Solomon et al., 1998[46]** | RCT | ■ | ■ | **‡** | ■ | **‡** | **‡** | ■ | ● | ● |

●: High risk. ‡: Unclear risk. ■: Low risk. RCT: Randomised Controlled Trial. CCT: Controlled Clinical Trial.
